# Case of Intra-Inguinal Hernia of Unusual Size, (Brought before the Parisian Medical Society, March, 1841)

**Published:** 1841-11-20

**Authors:** Daniel Brainard

**Affiliations:** Chicago, Illinois


### ORIGINAL COMMUNICATIONS.

*Case of Intra-Inguinal Hernia of unusual size,* (brought before the Parisian Medical Society, March, 1841.)

BY DANIEL BRAINARD, M. D.

To the Editors of the Medical Examiner.

Dec 11th, 1836, saw Asa W. Pollard, æt. 30, of stout frame, labouring under the following symptoms. Great tenderness and pain on pressing the abdomen, costiveness, hiccough, retching, and restlessness; pulse 125. In the left inguinal region there was a tumefaction extending from the pubis above the anterior superior spine of the ileum. This had existed, according to the statement of the patient, from birth, and he had for many years been subject to attacks of "colic." The present attack originated suddenly on the 3d of December, since which time he had been treated for "inflammation of the bowels." The swelling in the groin was attributed to the testicle; that organ having never descended into the scrotum. On a careful examination at this period, the diagnosis was hernia, which, having long been irreducible, had at length become strangulated.

The operation, too long deferred to afford a favourable chance of success, was immediately performed. The skin, superficial fascia, and cellular tissue being successively divided by an incision extending from the external inguinal ring upwards and outwards two inches and a half, the aponeurosis of the external oblique muscle was exposed, tense,—and slightly elevated. Dividing this, and the herniary sac beneath it, the extent and situation of the hernia were fully exposed. The testicle, of a florid hue, was placed in the canal in such a manner as completely to close the external ring, while the loop of small intestine, of a dark brown colour, passed upwards and outwards, beneath the aponeurosis of the external oblique, an inch and a half above the spine of the ileum. The stricture was sought for, and appeared to exist in the neck of the sac, and with the finger within it, could be freely moved about in different directions. This being divided, and the adherences of the protruded portions of intestine with the sac and with one another, separated, it was returned without difficulty within the abdomen. Enemas were administered, and some gas, with small portions of fecal matter discharged, but without relief to the patient. He died on the 12th; twenty-four hours after the operation.

On examination after death, the protruded portion of intestine was found within the abdomen, of a dark colour, but entire; the calibre of the intestine above it was greatly increased; below, it was contracted, while at the point of stricture it was almost obliterated. The peritoneum covering the small intestines was red throughout, and covered with portions of coagulated lymph.

This variety has been classed with the "intra-inguinal," or "hernia within the canal." but the term intra parietal would be more appropriate. It is believed that the cases on record in which this variety of hernia has obtained considerable size without appearing externally, are not numerous. They are noticed in these terms by Boyer:

"This kind of hernia has only been known and clearly described of late, but it had been observed by several practitioners, and particularly by J. L. Petit, who has spoken of it as follows: 'I have seen some of them situated beneath the aponeurosis of the external oblique muscle; so that the parts having pushed the peritoneum beyond the transverse and internal oblique muscles, and, being unable to pass the external ring, were reflected between the aponeurosis and internal oblique, forming a broad flat tumour.'" (Traité des Maladies Chirurgicales, tome viii. p. 227.) Samuel Cooper (Surgical Dictionary) says, "the tumour is small, for if the protrusion increases, the parts escape through the lower opening of the canal.

Exceptions, however, are on record. Thus Mr. Lawrence dissected a case in a female where the aponeurosis of the external oblique was distended by a swelling equal in bulk to two fists, while another portion of the sac, as large as an egg, projected through the ring. Hesselbach's 8th plate also represents a hernia within the canal of considerable size in a female."

*Chicago, Illinois, Nov. 4, 1841.*

